# Case Report: Management of Malignancy-Exacerbated Pemphigus Vulgaris During COVID-19 Pandemic

**DOI:** 10.3389/fmed.2021.708284

**Published:** 2021-08-11

**Authors:** Alberto Corrà, Francesca Cammelli, Lavinia Quintarelli, Giuseppe Barbato, Ornella Le Rose, Adele Salemme, Giovanni Di Zenzo, Francesco Coratti, Alice Verdelli, Cristina Aimo, Elena Biancamaria Mariotti, Beatrice Bianchi, Fabio Cianchi, Marzia Caproni

**Affiliations:** ^1^Section of Dermatology, Department of Health Sciences, University of Florence, Florence, Italy; ^2^Division of Gastrointestinal Surgery, Careggi University Hospital, Azienda Ospedaliero-Universitaria Careggi, Florence, Italy; ^3^Rare Diseases Unit, Section of Dermatology, Department of Health Sciences, Azienda USL Toscana Centro, University of Florence, European Reference Network-Skin Member, Florence, Italy; ^4^Section of Radiology, Unità Sanitaria Locale Toscana Centro, “Piero Palagi” Hospital, Florence, Italy; ^5^Molecular and Cell Biology Laboratory, Istituto Dermopatico dell'Immacolata - Istituto di Ricovero e Cura a Carattere Scientifico, Rome, Italy

**Keywords:** esophageal cancer, pemphigus vulgaris, rituximab, intravenous immunoglobulin, immunoblot, paraneoplastic pemphigus, COVID-19, Ivor Lewis esophagectomy

## Abstract

Pemphigus vulgaris is an intraepidermal autoimmune mucocutaneous blistering disease whose etiopathogenesis includes various trigger factors, i.e., drugs and malignancies. We present a case of malignancy-exacerbated pemphigus vulgaris which required a careful diagnostic process in order to rule out paraneoplastic pemphigus, along with the challenges posed by the need of treating both cutaneous and oncologic diseases. Possible post-operative complications post-poned the start of first-line immunosuppressive treatment of pemphigus. Moreover, the infective risks had to be minimized during the peak of the COVID-19 pandemic in Italy. Intravenous immunoglobulins were chosen as “bridge” therapy before the tumor surgical excision, followed by rituximab in post-operative phase.

## Introduction

Pemphigus is a potentially fatal autoimmune blistering disease which affects both mucous membranes and the skin. It is characterized by IgG autoantibodies targeting desmoglein 3 and/or desmoglein 1, which are molecules involved in the adhesion between keratinocytes. Antibody/antigen binding results in the loss of the adhesion functions of desmogleins and intraepidermal blistering. Pemphigus has been shown to occur following various triggers, including drugs and malignancies. Esophageal cancer is one of the leading malignant diseases and the sixth most common cause of cancer-related death worldwide (1). We wish to present a case of a patient with a mild pemphigus vulgaris (PV) dramatically exacerbated following a diagnosis of a solid tumor of the gastrointestinal tract.

## Case Report

An 80-year-old man was referred to our clinic due to a 6-year history of PV. He previously received treatments with systemic corticosteroids and immunosuppressants, including azathioprine and mycophenolate mofetil, both pre-maturely interrupted after 6 and 4 months, respectively, due to inefficacy. A cycle of rituximab, followed by two maintenance administrations 6 and 12 months later, had revealed effective in establishing a prolonged remission off-therapy for about 2 years. He had an episode of thrombophlebitis of the lower limbs 2 years earlier, for which he was on treatment with rivaroxaban. At the time of the first dermatological visit, the physical examination showed erosions mainly affecting the trunk and scalp. The Pemphigus Disease Activity Index (PDAI) was 7. Because of the low disease activity, a treatment with topical steroids was attempted.

**Figure 1:**
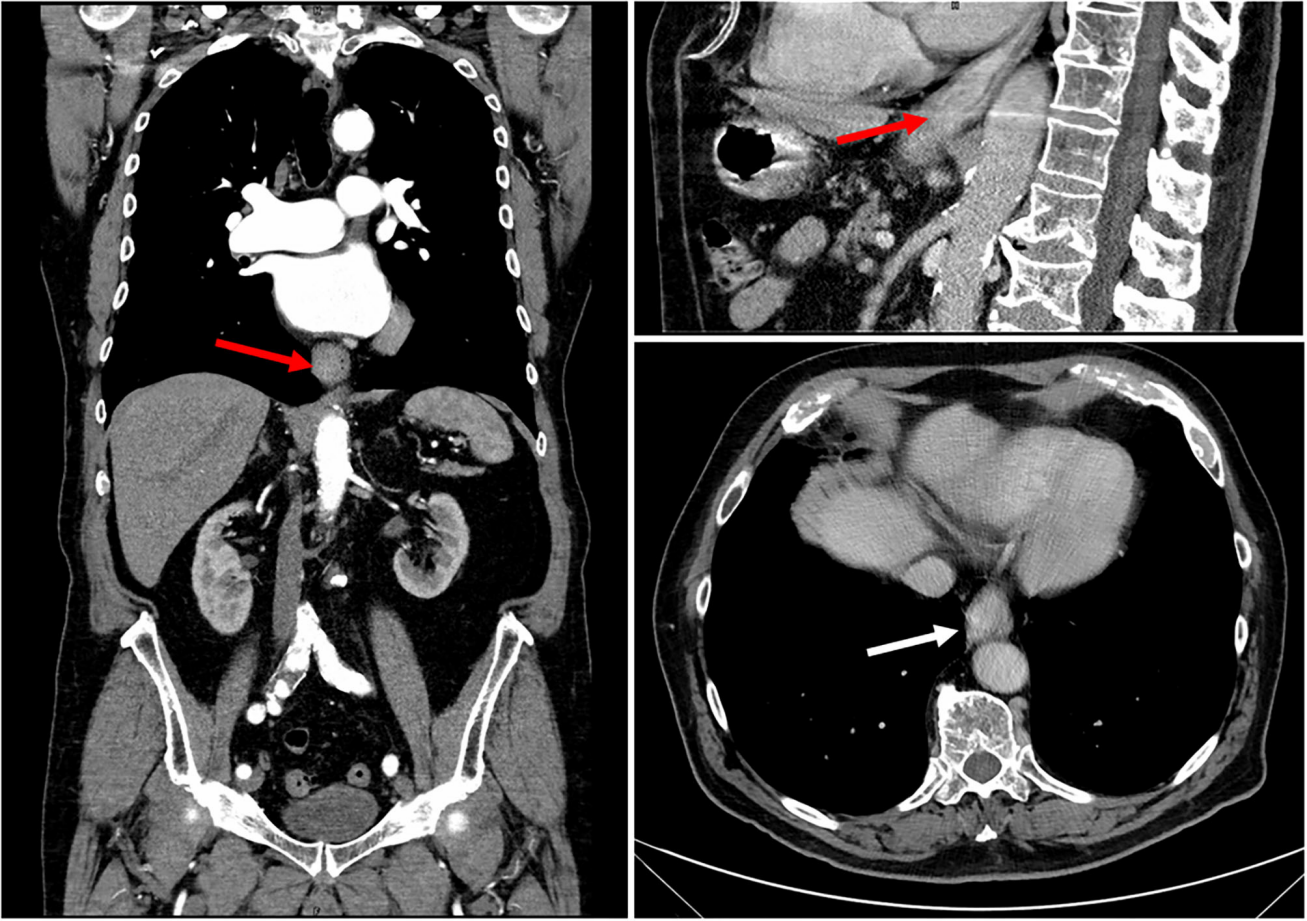
CT scan of the esophageal mass showing asymmetrical neoplastic wall thickening, extending from the lower third of esophagus to the cardia (white arrow in axial view and red arrows in sagittal and coronal views). The mass caused esophageal stenosis without dilatation of the upstream lumen.

After 4 weeks, the patient returned because of a significant worsening of his cutaneous lesions, which were enlarging and increasing in number, located in the head and neck, trunk, and limbs, with no mucosal sites involved. Pemphigus exacerbation was associated with the onset of other symptoms, including a progressive dysphagia, weight loss, and melena. A periodic re-assessment of serum autoantibody concentration also indicated a significant increase in both anti-Dsg1 and anti-Dsg3 antibody titers. An endoscopic examination of the distal esophagus indeed revealed a stenosing esophageal mass; a PET/CT scan confirmed the presence of a lesion 3 cm in diameter, highlighting metabolic activity areas corresponding only to tumor location, without nodal or distant metastasis (Figure 1). Previously, the chest RX and abdominal ultrasound conducted as screening for immunosuppressive treatments 2 years before were unremarkable, as well as the routine blood tests performed at that time.

**Figure 2:**
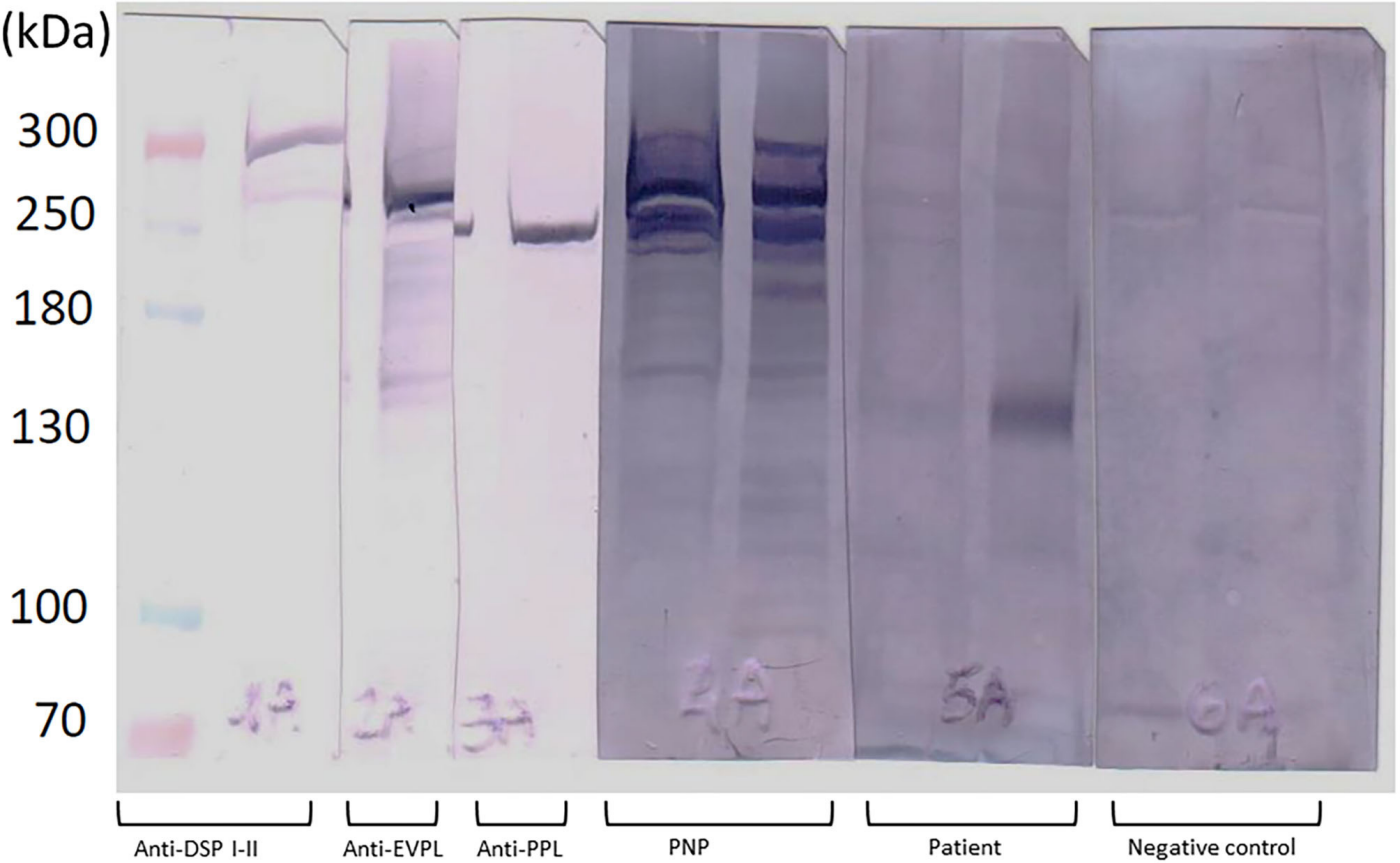
Results of immunoblot analysis on patient serum collected on week 7 (timepoint corresponding to the tumor diagnosis). The probe of the patient showed positivity for the 130-kDa band, corresponding to anti-DSG3 antibodies. No bands corresponding to anti-desmoplakin (anti-DSP I-II), anti-envoplakin (anti-EVPL), anti-periplakin (anti-PPL), or other autoantibodies specific for paraneoplastic pemphigus were detected in the analyzed serum. Double lanes for each serum: the first one corresponds to epidermal extracts prepared with 6 M urea, and the second one was for that prepared with 1% SDS.

The Gastrointestinal Surgery Unit opted for endoscopic resection of the tumor. In order to rule out paraneoplastic pemphigus (PNP), blood samples were collected for indirect immunofluorescence (IIF) on rat bladder and normal human skin and immunoblot for anti-plakin and anti-desmocollin autoantibodies, which resulted negative (Figure 2; Supplementary Figures 1–3).

Before the surgical intervention, delayed due to the COVID-19 outbreak, PV was managed with two cycles of intravenous immunoglobulins (IVIg, 2 g/kg per cycle) 4 weeks apart. The prolonged treatment with systemic steroids before major surgical interventions is associated with a significant risk of surgical complications; thereby, systemic steroids were not administered. After two cycles of IVIg treatment, the PDAI score remained stable.

The patient underwent robot-assisted Ivor–Lewis esophagectomy with latero-lateral anastomosis and jejunostomy feeding tube (J-tube) placement. Tissue perfusion and esophagogastric anastomosis evaluation with the indocyanine green method were performed. After the surgery, the patient achieved early mobilization, with no signs of anastomotic leak at endoscopies and CT scan. The post-operative course was uneventful, except for an episode of atrial fibrillation, which was managed with two subsequent doses of verapamil according to the advice of the cardiologist. The patient was then discharged on post-operative day 11, with no dysphagia nor nausea reported. The histopathologic examination of the esophageal mass confirmed the diagnosis of gastric cardia adenocarcinoma.

**Figure 3:**
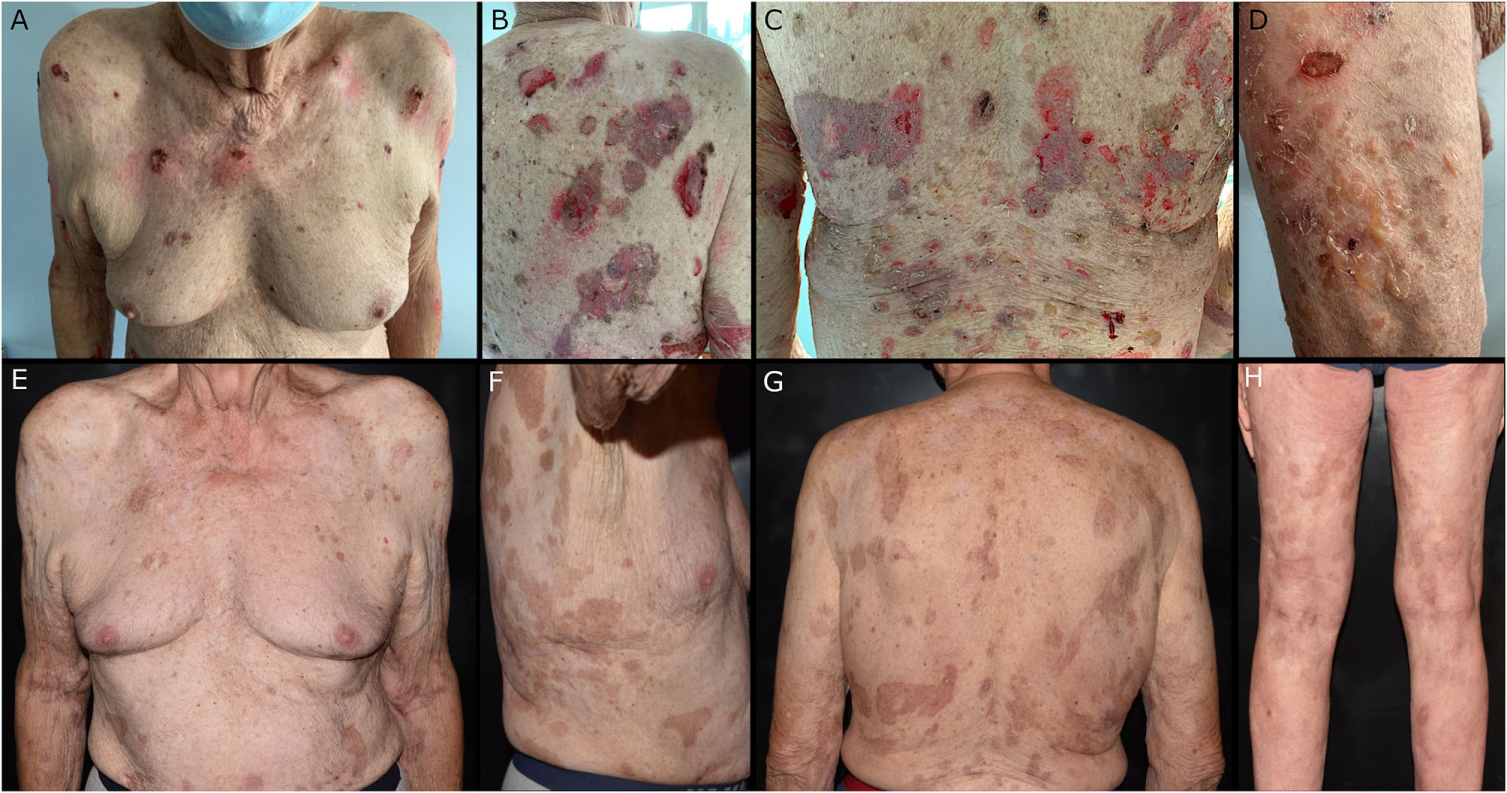
**(A–D)** Soft blisters and painful erosion affecting the trunk and limbs a few days after the surgical removal of the esophageal mass. **(E–H)** Residual post-inflammatory hyperpigmentation in sites previously affected by pemphigus lesions at the 4-month follow-up.

Despite the successful surgical management of the tumor, PV continued to worsen, with blisters and erosions affecting the entire body, except for mucosal sites and feet (Figure 3).

Considering the recommendation to avoid high-dose systemic steroids also after surgery, due to the risk of anastomotic leakage, the patient was managed with 4 weekly infusions of rituximab at a dose of 500 mg (lymphoma protocol). A moderate-dose systemic steroid therapy (prednisone 0.6 mg/kg/day) was started only 2 weeks later in concomitance with the end of the national lockdown.

**Figure 4:**
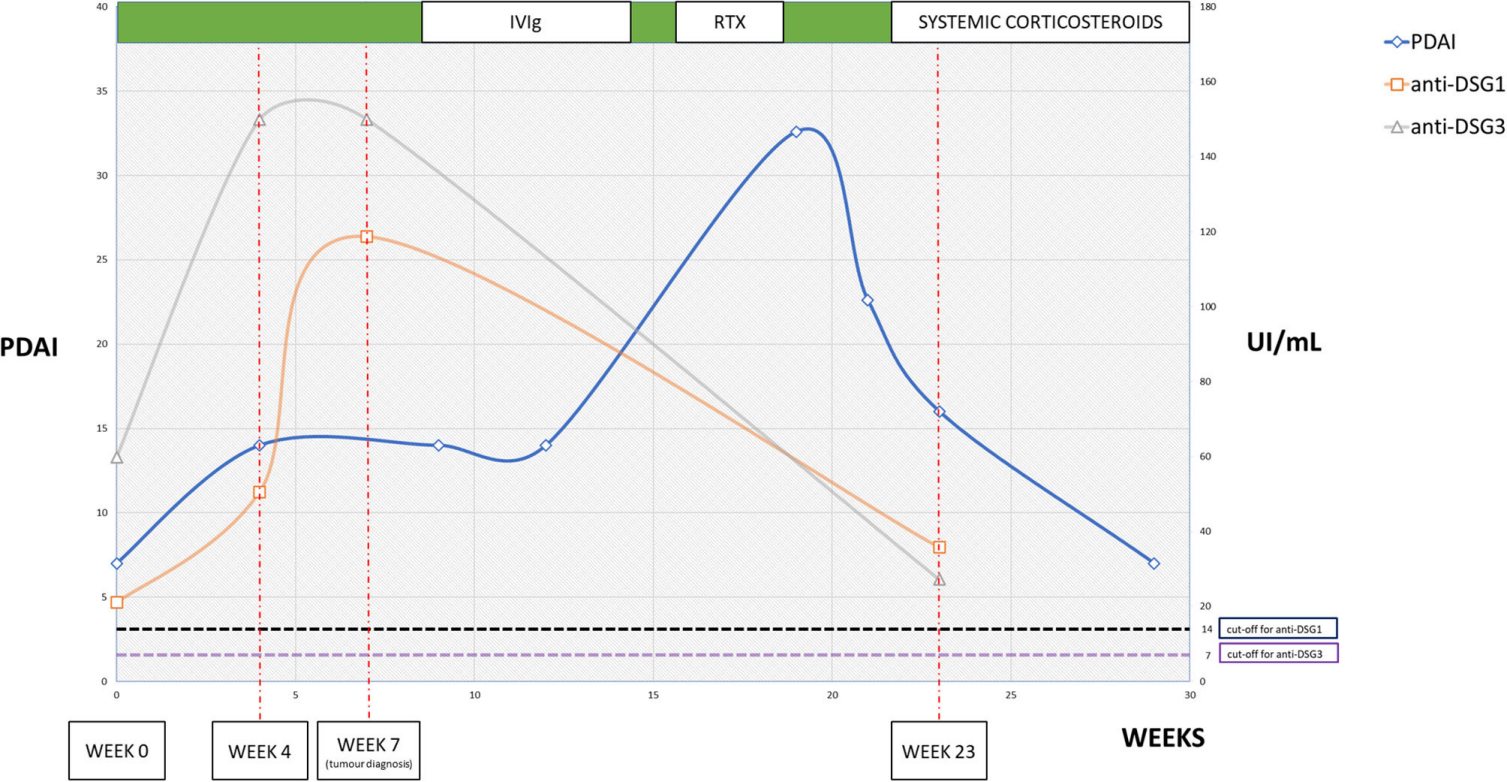
Graph showing the trend of Pemphigus Disease Activity Index score and anti-desmoglein serum titer. Week 0 is considered the time of onset of gastrointestinal symptoms. Week 4: clinical worsening and increase in anti-desmoglein titers. Week 7: diagnosis of esophageal mass. Week 9: end of the first IvIG cycle. Week 13: end of the second IvIG cycle. Week 16: first rituximab infusion. Week 19: end of rituximab therapy. Week 21: start of prednisone, 0.6 mg/kg/day. Week 23: clinical follow-up and re-assessment of anti-desmoglein titers. The titers seemed to predict the clinical worsening of the disease, which remained stable during the period of IvIG infusions. The worsening following the surgical intervention showed a prompt response to rituximab. The timing of single treatments is indicated above.

The skin of the patient started improving progressively, with complete resolution of the lesions. The anti-DSG1 and anti-DSG3 titers likewise decreased progressively (Figure 4).

At the 4-month dermatologic follow-up, only post-inflammatory hyperpigmentation or hypopigmentation was detectable, with no active lesions (Figure 3), while the last enzyme-linked immunosorbent assay (ELISA) performed resulted in lower anti-desmoglein autoantibody concentrations. No surgery-related criticalities or tumor recurrences were highlighted during this period.

## Methods

### Anti-Desmoglein Enzyme-Linked Immunosorbent Assay

Assessments of circulating anti-DSG1 and anti-DSG3 autoantibodies were performed through ELISA using commercial kits (MBL MESACUP-2 TEST, Naka-Ku Nagoya Aichi, Japan). The kit was composed of microwells which were coated with recombinant DSG1 and DSG3. The cutoff values for positivity were 14 UI/ml for anti-DSG1 and 7 UI/ml for anti-DSG3, according to the indication of the manufacturer.

### Envoplakin ELISA

Assessment of circulating anti-envoplakin autoantibodies was performed through ELISA using commercial kits (Euroimmun, Lubeck, Germany), according to the protocol of the manufacturer. The cutoff values for envoplakin positivity was 18.6 UI.

### Indirect Immunofluorescence

IIF on rat bladder was performed using a commercial kit (FA 1507-1005, EUROIMMUN AG, Lübeck, Germany) containing rat bladder slides and fluorescein isothiocyanate-conjugated anti-human IgG secondary antibodies and then examined under a Nikon C2 confocal microscope. Indirect Immunofluorescences were also performed on normal human skin substrate with patient sera collected at timepoints week 0, 4, 7, and 23.

### Immunoblot

SDS-polyacrylamide gel electrophoresis was performed using 6% polyacrylamide gel loaded with epidermal extracts prepared with 6 M urea (left lane) and 1% SDS (right lane) for patient and control sera, and epidermal extracts were prepared with urea for control antibodies in reducing conditions. After transfer to polyvinylidene difluoride membrane (Immobilon-P, Millipore, USA) and blocking in 5% non-fat milk (Merck, Germany), immunoreactivity was detected by incubation with 1:20 and 1:100 dilution of patient and control sera and antibodies to Desmoglein 1 (Santa Cruz, California), Desmoglein 3 (R&D, Minneapolis), Desmocollin 1 (Progen, Germany), Desmocollin 2 (Progen, Germany), Desmoplakin I and II (Progen, Germany), Envoplakin (Santa Cruz, California), and Periplakin (Santa Cruz, California). After washing, the membrane was incubated with alkaline phosphatase-labeled secondary antibody against IgG (Southern Biotech, USA and CA). Then, it was washed again and stained with chromogenic substrates, 5-bromo-4-chloro-3-indolyl-phosphate and nitroblue tetrazolium (Roche, Swiss).

## Results

Different ELISAs for detecting anti-DSG1 and anti-DSG3 autoantibodies were performed (Figure 4). At week 0, the anti-DSG1 titer was 21.1 UI/ml and the anti-DSG3 titer was 59.9 UI/ml. At week 4, the values were significantly increased as follows: 50.6 UI/ml for anti-DSG1 and >150 UI/ml for anti-DSG3. At week 7, the anti-DSG1 was 118.7 UI/ml, while the anti-DSG3 was >150 UI/ml. The values then decreased to 35.8 UI/ml for anti-DSG1 and 27.2 UI/ml for anti-DSG3 by week 23. The ELISA for envoplakin was performed on the same sera collected and showed negative results, as shown in Supplementary Figure 1B.

No staining was visible with a confocal microscope on rat bladder indirect immunofluorescence performed on weeks 0, 4, 7, and 23 sera (Supplementary Figure 3).

Patient sera showed positivity for autoantibodies targeting the 130-kDa band on immunoblot analysis, which corresponds to DSG3. No reactivity at bands corresponding to plakin family or desmocollin family proteins was noted (Figure 4; Supplementary Figure 1A). The investigations were performed on serum collected at weeks 0, 4, 7, and 23.

## Discussion

This is a case of PV worsened in concomitance with the development of a solid gastrointestinal tumor. Pemphigus diagnosis was made several years before the detection of the neoplasm, which therefore may represent an exacerbating factor for the bullous disease rather than of inductor. Relapses of PV after rituximab treatment are not infrequent, involving up to 63% of patients even years after a single cycle of therapy, while the rate seems to be low in patients also treated with adjunctive maintenance rituximab administrations (2). However, the timing raised the suspicion of PNP (3), an autoimmune mucocutaneous disease for which Nguyen et al. in 2001 proposed the term “paraneoplastic autoimmune multiorgan syndrome” (PAMS), in consideration of the frequent multi-systemic involvement (4). Although the correlation with malignancy seemed to be clear, PNP/PAMS diagnosis was excluded for different reasons. Mucosal and respiratory tract involvement was completely absent, while skin lesions resembled a classical PV blister and erosions. Moreover, IIF on rat bladder revealed repeatedly negative results, as well as immunoblot for anti-envoplakin and anti-desmocollin autoantibodies. Serological analysis was performed on sera collected at different timepoints (Supplementary Figure 1): week 0 is considered the time of onset of tumor-related symptoms, week 4 consists in the subsequent patient visit, week 7 is the time of the tumor diagnosis, and week 23 represents the first follow-up after the rituximab cycle (Figure 4). The correlation between PV and malignancies is well-established, with malignancies reported in showing a role in induction or exacerbation of a previously diagnosed PV (5, 6). Hematologic malignancies seem to have a higher incidence among PV patients (7, 8), but a large-population study also evidenced the association with oropharyngeal and colon carcinomas, which were present in 0.9 and 3.7% of PV patients, respectively. In general, gastrointestinal cancers were present in 6.1% of patients, compared to the 2.8% of the control population. In addition, a large population-based study found a significant association between PV and esophageal cancer (9). The patient described in this case had a history and clinical presentation consistent with PV, considering the lack of severe mucosal involvement and serological investigations. Despite the serological positivity for both anti-Dsg1 and anti-Dsg3 IgG, the patient never showed mucosal involvement, while the cutaneous lesions were clinically more compatible with PV than with pemphigus foliaceous. Interestingly, other authors hypothesized the existence of a “cutaneous-type” PV, describing similar cases where the absence of mucosal lesions could be due to the low pathogenetic activity of anti-Dsg3 not sufficient to induce blistering as in the mucocutaneous or mucosal-dominant phenotypes of PV (10, 11).

The management of the patient required an urgent surgical excision of the neoplasm due to the symptoms triggered and the potentially life-threatening course of the disease. Pemphigus-specific immunosuppressive treatments had to be post-poned in order to avoid perioperative risks and infective complications. In fact, a prolonged high-dosed systemic steroid therapy could have a detrimental effect on the healing process of anastomosis, raising the leakage risk (12, 13). Moreover, the concomitant peak of COVID-19 pandemic in Italy emphasized the need to reduce the infective risk. Thus, surgeons and dermatologists temporarily post-poned the anti-CD20 therapy to reduce the level of immunosuppression. The choice of administering IVIg was guided by the evidence of effectiveness in both pemphigus and COVID-19 disease (14, 15).

This case highlights the possibility of hidden neoplasms in patients experiencing a severe and sudden worsening of pemphigus. The physiopathology of this phenomenon is still unknown and is probably a complex relationship which needs further studies. The association between cancer and autoimmunity is bidirectional, as different autoimmune disorders can promote tumorigenesis; malignancies may likewise increase the risk of autoimmune diseases (16). Tissues affected by an autoimmune disease may present localized reduction of self-tolerance, leading to chronic inflammation and oncogenesis (17). On the other hand, neoantigens expressed early in cancer may elicit an immune response, enhancing autoimmune disease activity indirectly through B cell maturation and proliferation with increased autoantibody production.

It is clear that the generation of many abnormal antigens during tumorigenesis can be recognized early by the immune system, thus triggering a reaction known as cancer immunoediting, which can induce the host immune response to produce autoantibodies in the early phase before the clinical manifestations. This induced researchers to investigate these autoantibodies as early diagnostic biomarkers for esophageal squamous cell carcinoma and esophagogastric junction adenocarcinoma (18).

Considering the possible association between pemphigus and cancer may lead to an earlier tumor diagnosis, thus influencing the survival rate and treatment regimen. The management of severe PV in oncologic patients poses significant challenges due to the risk of immunosuppressive treatments and absence of dedicated guidelines. Our patient required rituximab therapy due to the lack of sufficient disease control by IVIg treatment and the need to avoid a high dose of systemic corticosteroids. The high efficacy of rituximab in the short term counterbalanced the risk of tumor progression with prolonged B-cell depletion. A close collaboration between dermatologists, surgeons, and oncologists is needed to adopt the best treatment strategy in order to improve patient outcome and quality of life.

## Perspective of the Patient

The patient was obviously scared about the diagnosis of esophageal adenocarcinoma and afraid of the possibility of dying soon. Subsequently, the worsening of pemphigus caused the well-known sensations of burning and extreme pain in a large part of the total skin surface, impairing his quality of life and the normal activities of daily living. During the treatment with IVIg, the distress was so intense that the patient said repeatedly that it was the most painful sensation of his life. Besides that, the risk of tumor progression and the known low survival rate of advanced esophageal cancer convinced him to undergo the surgical intervention, along with the awareness of the slow progression of symptoms which could lead to the incapability of feeding.

The successful surgical intervention represented a strong signal for him, particularly due to the early discharge with prompt recovery of normal alimentation. The positivity derived from this part of the therapeutic course allowed him to face the treatment with rituximab with more determination, focusing on the possibility to get a full recovery.

## Data Availability Statement

The original contributions generated for the study are included in the article/Supplementary Material, further inquiries can be directed to the corresponding author.

## Ethics Statement

Written, informed consent was obtained from the patient for the publication of this case report.

## Author Contributions

MC, AC, LQ, AV, CA, and MM were directly involved in patient management, produced and drafted the manuscript and revisioned the literature. MC concepted the whole work and revised carefully the manuscript. OL collected radiological images and revised the manuscript. FCa, GB, FCo, and FCi were involved in the surgical aspect of patient management, revised the manuscript, and collected histopathological pictures. AS, GD, and BB provided immunoserological analysis, collected the images of immunoblot, and indirect immunofluorescence. All authors contributed to the article and approved the submitted version.

## Conflict of Interest

The authors declare that the research was conducted in the absence of any commercial or financial relationships that could be construed as a potential conflict of interest.

## Publisher's Note

All claims expressed in this article are solely those of the authors and do not necessarily represent those of their affiliated organizations, or those of the publisher, the editors and the reviewers. Any product that may be evaluated in this article, or claim that may be made by its manufacturer, is not guaranteed or endorsed by the publisher.

## Supplementary Material

Supplementary Figure 1 Serological tests for patient diagnosis. **(A)** Immunoblotting on epidermal extracts did not show any significant (absent in controls) bands for PNP diagnosis. Lane 1, anti-desmoglein 1 (160 kDa); lane 2, anti-desmoglein 3 (130 kDa); lane 3, anti-desmocollin 1 (110 kDa); lane 4, anti-desmocollin 2 (120 kDa); lane 5, anti-envoplakin (210 kDa); lane 6, anti-periplakin (190 kDa); lane 7, anti-desmoplakin 1/2 (250 and 210 kDa); lane 8, paraneoplastic pemphigus serum (positive control); lanes 9–12, sera of the patient at weeks 0, 4, 7, and 23, respectively; lanes 13–15, negative control sera. **(B)** Sera from the present case did not react with envoplakin by ELISA. Paraneoplastic pemphigus serum (positive control) and sera of the patient at weeks 0, 4, 7, and 23 were tested by envoplakin ELISA. Cutoff value = 18.6 units (dashed line).

Supplementary Figure 2 Indirect immunofluorescence on normal human skin performed on patient serum collected at weeks 0, 4, 7, and 23. The sera were tested for both IgG and IgA at each timepoint with 1:20 dilution for IgG and 1:10 for IgA. The pictures show mild–moderate positive staining for IgG, while IgA was negative in all timepoints.

Supplementary Figure 3 Indirect immunofluorescence performed on rat bladder substrate at the same timepoints (weeks 0, 4, 7, and 23). All the probes resulted negative compared to the positive control.

Supplementary Figure 4 Histological pictures of adenocarcinoma of the gastro-esophageal junction. **(A)** Tubular and solid pattern gastric adenocarcinoma infiltrating submucosal tissue (magnification ×100, hematoxylin and eosin). **(B)** Lauren intestinal-type gastric adenocarcinoma; infiltrative glands with different (moderate and low) grades of differentiation (original magnification ×200, hematoxylin and eosin).
