# Supplementary figures and images for: Case Report: Management of Malignancy-Exacerbated Pemphigus Vulgaris During COVID-19 Pandemic
Source: Front Med (Lausanne). 2021 Aug 11;8:708284. doi: 10.3389/fmed.2021.708284 (PMC8385714; doi:10.3389/fmed.2021.708284)

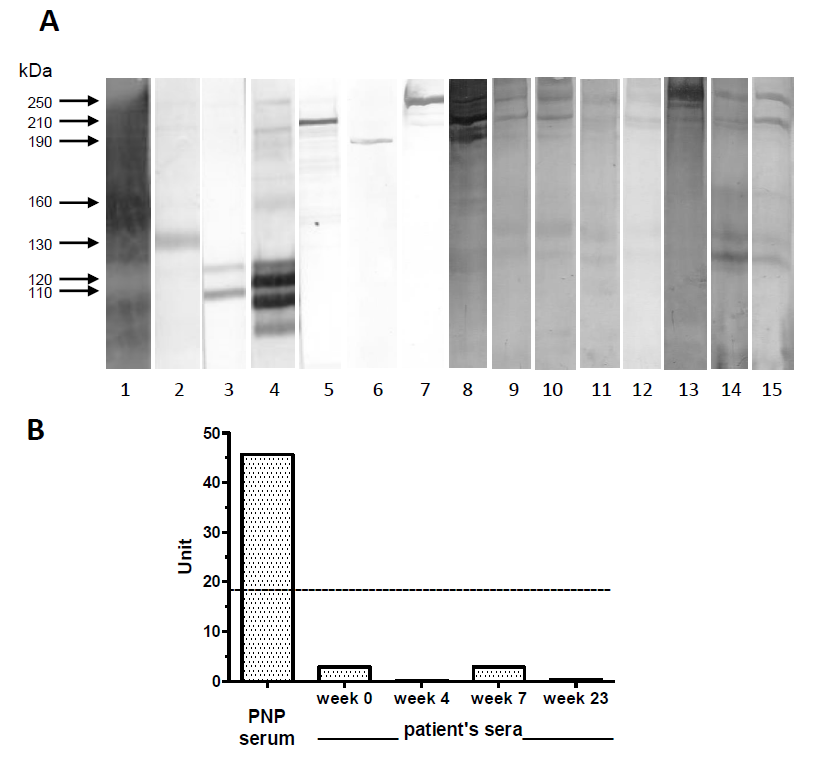

Supplement: Supplementary Figure 1 — Serological tests for patient diagnosis. (A) Immunoblotting on epidermal extracts did not show any significant (absent in controls) bands for PNP diagnosis. Lane 1, anti-desmoglein 1 (160 kDa); lane 2, anti-desmoglein 3 (130 kDa); lane 3, anti-desmocollin 1 (110 kDa); lane 4, anti-desmocollin 2 (120 kDa); lane 5, anti-envoplakin (210 kDa); lane 6, anti-periplakin (190 kDa); lane 7, anti-desmoplakin 1/2 (250 and 210 kDa); lane 8, paraneoplastic pemphigus serum (positive control); lanes 9–12, sera of the patient at weeks 0, 4, 7, and 23, respectively; lanes 13–15, negative control sera. (B) Sera from the present case did not react with envoplakin by ELISA. Paraneoplastic pemphigus serum (positive control) and sera of the patient at weeks 0, 4, 7, and 23 were tested by envoplakin ELISA. Cutoff value = 18.6 units (dashed line). [file Image_1.TIF]

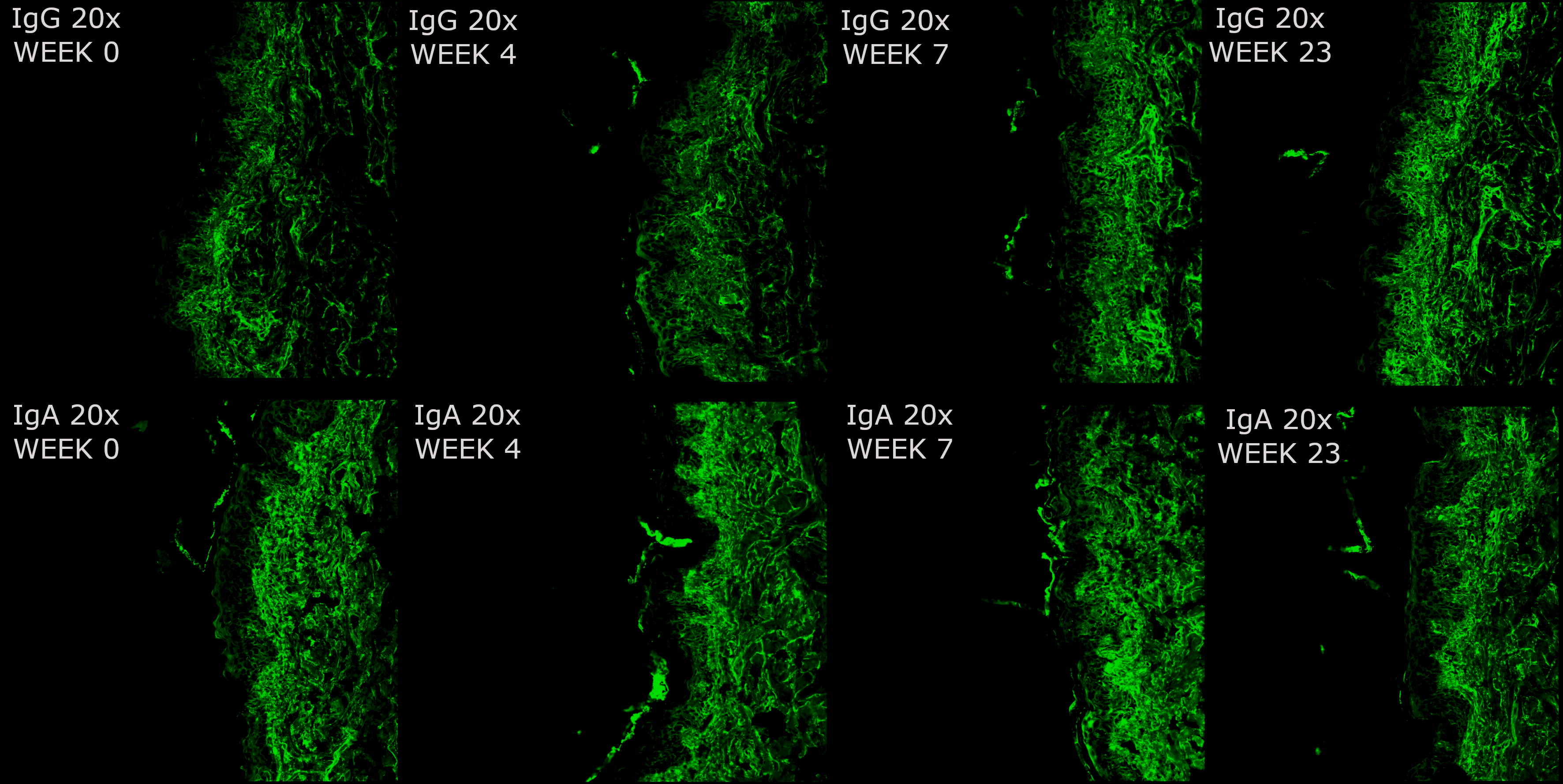

Supplement: Supplementary Figure 2 — Indirect immunofluorescence on normal human skin performed on patient serum collected at weeks 0, 4, 7, and 23. The sera were tested for both IgG and IgA at each timepoint with 1:20 dilution for IgG and 1:10 for IgA. The pictures show mild–moderate positive staining for IgG, while IgA was negative in all timepoints. [file Image_2.TIF]

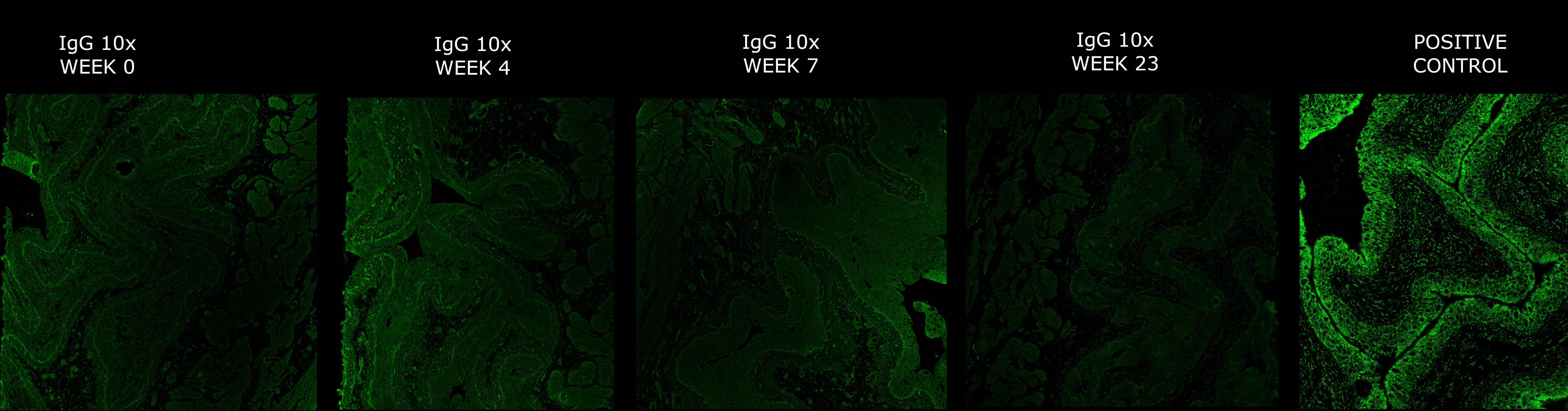

Supplement: Supplementary Figure 3 — Indirect immunofluorescence performed on rat bladder substrate at the same timepoints (weeks 0, 4, 7, and 23). All the probes resulted negative compared to the positive control. [file Image_3.JPEG]

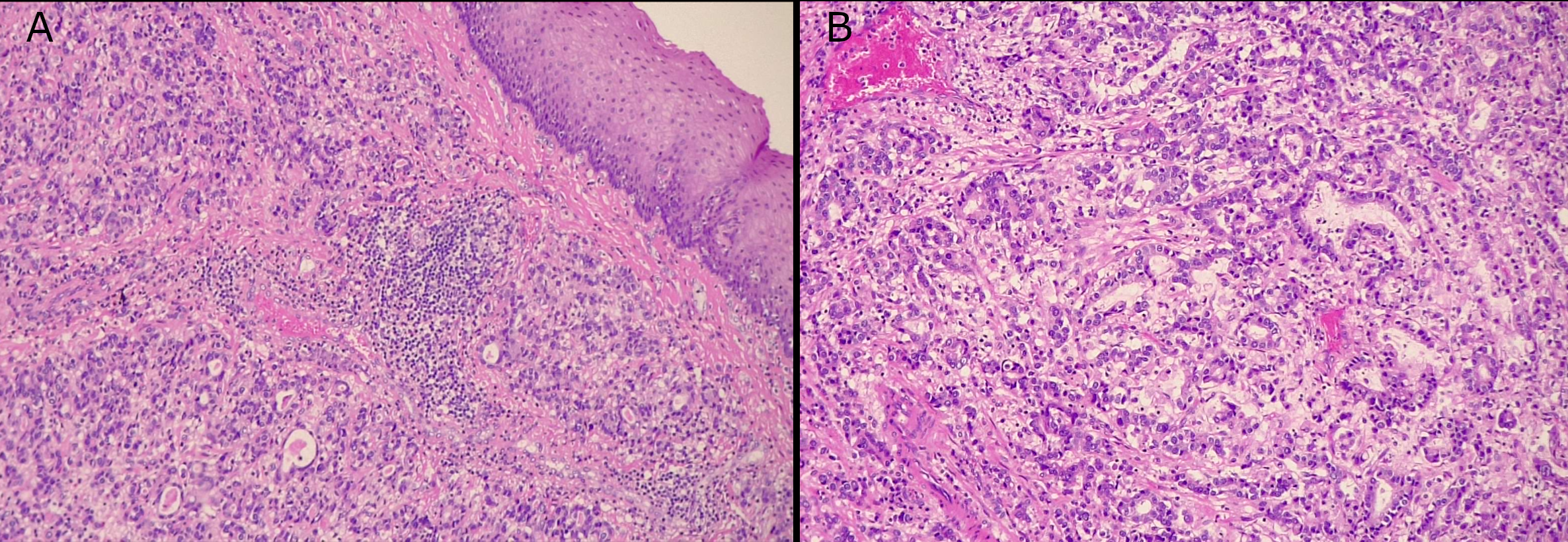

Supplement: Supplementary Figure 4 — Histological pictures of adenocarcinoma of the gastro-esophageal junction. (A) Tubular and solid pattern gastric adenocarcinoma infiltrating submucosal tissue (magnification ×100, hematoxylin and eosin). (B) Lauren intestinal-type gastric adenocarcinoma; infiltrative glands with different (moderate and low) grades of differentiation (original magnification ×200, hematoxylin and eosin). [file Image_4.TIF]
